# Supplementary figures and images for: Systematic analysis of JmjC gene family and stress­-response expression of KDM5 subfamily genes in Brassica napus
Source: PeerJ. 2021 Mar 31;9:e11137. doi: 10.7717/peerj.11137 (PMC8019318; doi:10.7717/peerj.11137)

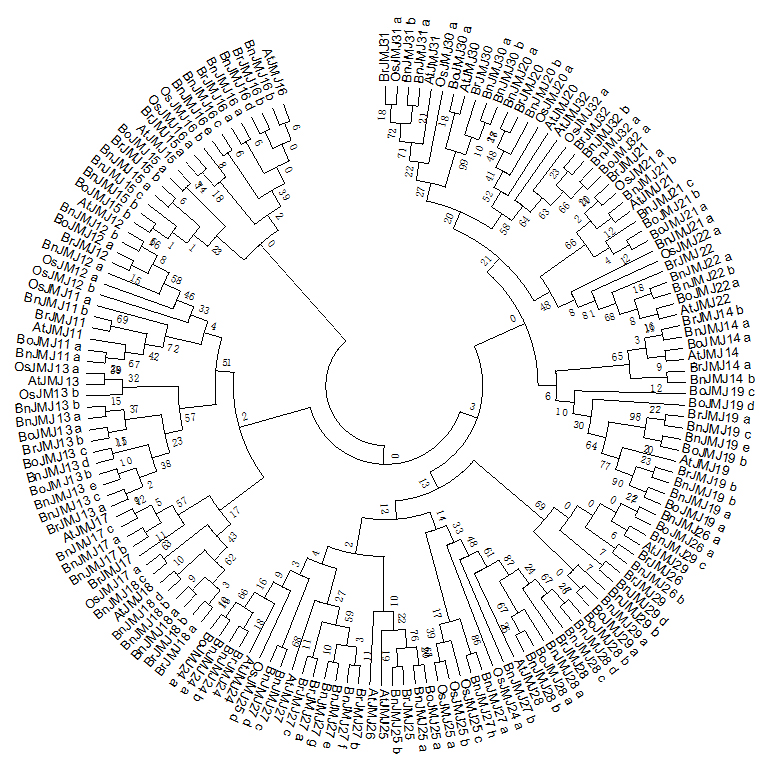

Supplement: Supplemental Information 3 — ML phylogenetic tree is constructed based on the same sequence of NJ-phylogenetic tree. The phylogenetic tree analysis was performed using MEGA7 with the following settings: tree inference as Nearest-Neighbor-Interchange (NNI); include sites as complete deletion option for total sequences analysis. [file peerj-09-11137-s003.jpg]
